# Molecular characterization of *Cryptosporidium* spp. from patients with diarrhoea in Lusaka, Zambia

**DOI:** 10.1051/parasite/2020050

**Published:** 2020-10-13

**Authors:** Namwiinga Rozaria Mulunda, Kyoko Hayashida, Junya Yamagishi, Sandie Sianongo, Gilbert Munsaka, Chihiro Sugimoto, Mable Mwale Mutengo

**Affiliations:** 1 Department of Pathology and Microbiology, University Teaching Hospital 10101 Lusaka Zambia; 2 Division of Collaboration and Education, Hokkaido University Research Center for Zoonosis Control 001-0020 Sapporo Japan; 3 International Collaboration Unit, Research Center for Zoonosis Control, Hokkaido University 001-0020 Sapporo Japan; 4 Institute of Basic and Biomedical Sciences, Levy Mwanawasa Medical University Great East Road 10101 Lusaka Zambia

**Keywords:** *Cryptosporidium*, Diarrhoeal disease, Zambia, Subtype, Zoonosis

## Abstract

*Cryptosporidium* is a major etiological agent of diarrhoeal diseases among children and immune-compromised individuals in sub-Saharan African countries. We conducted a study to determine the prevalence and genetic characteristics of *Cryptosporidium* spp. in stool samples from patients with diarrhoea who presented at the University Teaching Hospital in Lusaka, Zambia. *Cryptosporidium* species and subtypes from 71 microscopically confirmed cryptosporidiosis stool samples collected between 2017 and 2019 were determined by polymerase chain reaction followed by partial sequencing of the small subunit rRNA and 60-kDa glycoprotein (*gp60*) gene. Additionally, data for the period between 2014 and 2019 were reviewed and analysed for cryptosporidiosis seasonal and age distribution. *Cryptosporidium* was more prevalent in the rainy season. The highest number of cases was reported among the 1–4 year age group. By sequence analysis of the 71 positive isolates, *Cryptosporidium hominis* (*n* = 42; 59.2%), *C. parvum* (*n* = 27; 38%), *C. felis* (*n* = 1; 1.4%), and *C. meleagridis* (*n* = 1; 1.4%) were identified. Four *C. hominis* subtype families (Ia, Ib, Id, and Ie) and three *C. parvum* subtype families (IIc, IIe, and IIs) were identified. The most frequent subtypes were IeA11G3T3 (*n* = 20; 28.2%), IIcA5G3 (*n* = 12; 16.9%), IIeA12G1 (*n* = 11; 15.5%) and IaA30R3 (*n* = 10; 14.1%). The observed species/subtypes of *C. hominis* and *C. parvum* indicated that the infection was mainly transmitted through the anthroponotic route. The identification of *C. felis* and *C. meleagridis* suggests that an atypical zoonotic transmission cycle also exists.

## Abstract (fr)

*Cryptosporidium* est un agent étiologique majeur des maladies diarrhéiques chez les enfants et les personnes immunodéprimées dans les pays d’Afrique subsaharienne. Nous avons mené une étude pour déterminer la prévalence et les caractéristiques génétiques de *Cryptosporidium* spp. dans des échantillons de selles de patients souffrant de diarrhée qui se sont présentés à l’hôpital universitaire de Lusaka, en Zambie. Les espèces et sous-types de *Cryptosporidium* provenant de 71 échantillons de selles de cryptosporidiose, confirmés au microscope et prélevés entre 2017 et 2019, ont été déterminés par réaction en chaîne par polymérase suivie d’un séquençage partiel de la petite sous-unité de l’ARNr et du gène de la glycoprotéine de 60 kDa (gp60). De plus, les données pour la période entre 2014 et 2019 ont été examinées et analysées pour la distribution saisonnière et par âge de la cryptosporidiose. *Cryptosporidium* était plus répandu pendant la saison des pluies. Le plus grand nombre de cas a été signalé dans le groupe d’âge de 1 à 4 ans. Par analyse séquentielle des 71 isolats positifs, *Cryptosporidium hominis* (*n* = 42 ; 59,2 %), *C. parvum* (*n* = 27 ; 38 %), *C. felis* (*n* = 1 ; 1,4 %) et *C. meleagridis* (*n* = 1 ; 1,4 %) ont été identifiés. Quatre familles de sous-types de *C. hominis* (Ia, Ib, Id et Ie) et trois familles de sous-types de *C. parvum* (IIc, IIe et IIs) ont été identifiées. Les sous-types les plus fréquents étaient A11G3T3 (*n* = 20 ; 28,2 %), IIcA5G3 (*n* = 12 ; 16,9 %), IIeA12G1 (*n* = 11 ; 15,5 %) et IaA30R3 (*n* = 10 ; 14,1 %). Les espèces/sous-types observés de *C. hominis* et *C. parvum* indiquent que l’infection est principalement transmise par voie anthroponotique. L’identification de *C. felis* et *C. meleagridis* suggère qu’il existe également un cycle de transmission zoonotique atypique.

## Introduction

*Cryptosporidium* is one of the most prevalent protozoan parasites causing diarrhoea in humans, but it has been largely neglected [29]. It is a major cause of diarrhoeal diseases in children in developing countries and is associated with occasional outbreaks among adults in developed countries [6, 24]. *Cryptosporidium* is also an opportunistic parasite, which has been associated with diarrhoea in immune-compromised individuals and is a major cause of morbidity and mortality among such populations [26, 28, 30]. In immune-competent individuals, the infection is either asymptomatic or may cause self-limiting diarrhoea that normally resolves within a week or two [26, 28]. However, in immune-compromised individuals or children, diarrhoea is usually severe and life-threatening, lasting for more than two weeks if not effectively managed [28]. Cryptosporidiosis is usually associated with poor hygiene and is transmitted through contaminated food or water via the fecal-oral route [30].

More than 30 species of *Cryptosporidium* have been identified worldwide, with *C. hominis* and *C. parvum* accounting for most of the infections in humans [36]. *Cryptosporidium hominis* primarily infects humans, whereas *C. parvum* has both zoonotic and anthroponotic genotypes [30, 36]. These species can be further classified into subtype families and subtypes based on the 60 kDa glycoprotein (*gp60*) gene sequence. Some subtype families such as *C. parvum* IIc and IIe are predominantly found in humans, especially in lower-income countries, and are considered to be anthroponotic [17, 37]. Other less common *Cryptosporidium* species that are primarily animal infective pathogens, but that can also occasionally infect humans include *C. meleagridis*, *C. felis*, *C. canis*, *C. ubiquitum*, *C. cuniculus*, *C. viatorum*, and *C. muris* [9].

While *Cryptosporidium* spp. have been documented from both asymptomatic and symptomatic people in Zambia [27, 32, 33], data on subtype characterisation of the species are limited. In this study, we aimed to document *Cryptosporidium* species and subtypes circulating in patients with diarrhoea at the University Teaching Hospital in Lusaka, Zambia. Additionally, the prevalence and seasonal distribution of *Cryptosporidium* were determined.

## Materials and methods

### Ethics statement

This study was approved by the University of Zambia Biomedical Research Ethics Committee (IRB00001131). Further clearance to conduct this study and publish the findings was given by the National Health Research Authority.

### Study population and *Cryptosporidium* sample collection

This study was conducted at the University Teaching Hospital (UTH), a tertiary-level government hospital located in Lusaka, Zambia. Data for 19,033 patients who had submitted stool samples for routine parasitological investigations over 5 years (January 2014–May 2019) were reviewed and analysed. The cryptosporidiosis case prevalence, age, and seasonal distribution were determined. Fisher’s exact test was used to determine differences in the prevalence of cryptosporidiosis by age-group and sex. A *p*-value below 0.05 was considered statistically significant. Monthly precipitation data at Lusaka airport during the study period were obtained from SASSCAL WeatherNet (www.sasscalweathernet.org). All 19,033 samples were routinely examined for *Cryptosporidium* oocysts by microscopic examination of modified Ziehl–Neelsen-stained stool smears [13]. Between 2017 and 2019, 71 microscopically confirmed *Cryptosporidium* spp.-positive faecal samples were stored at −20 °C before the molecular characterisation of species and subtypes. Detection of other parasites was done using the formol-ether concentration method. Briefly, about 1 g of stool was mixed with 10% formalin and was sieved through gauze into a centrifuge tube to which 3 mL of ether were added. After vigorous mixing, it was centrifuged at 3000 rpm, as previously described [1], and the deposit was microscopically examined for the presence of intestinal parasites.

### DNA isolation and small subunit (SSU) rRNA- and *gp60*-polymerase chain reaction (PCR)

DNA from approximately 150 mg of each faecal sample was extracted from 71 *Cryptosporidium-*positive samples using a ZR fecal DNA mini kit (Zymo Research, Orange, CA, USA), after three cycles of freeze-thawing. The eluted DNA was transferred to a clean sterile 1.5 mL microcentrifuge tube and was stored at −20 °C until use in downstream molecular analysis. To characterise *Cryptosporidium* species and subtypes, nested PCR for both SSU rRNA and 60 kDa glycoprotein (*gp60*) genes were performed. For the SSU rRNA gene, primer sets SSU F1 (5′–TTCTAGAGCTACATGCG–3′) and SSU R1 (5′–CCCATTTCCTTCGAAACAGGA–3′) for the primary reaction, and SSU F2 (5′–GGAAGGGTTGTATTTATTAGATAAAG–3′) and SSU R2 (5′–CTCATAAGGTGCTGAAGGAGTA–3′) for the secondary reaction were used [38]. The *gp60* PCR primer sets consisted of AL3531 (5′–ATAGTCTCCGCTGTATTC–3′) and AL3535 (5′–GGAAGGAACGATGTATCT–3′) for the primary reaction and AL3532 (5′–TCCGCTGTATTCTCAGCC–3′) and AL3534 (5′–GCAGAGGAACCAGCATC–3′) for the secondary reaction [2]. The mixture for both reactions consisted of 5 μL of Ampdirect buffer (Shimadzu, Kyoto, Japan), 0.75 μL of each 10 μM primer, 0.1 μL of BIOTAQ DNA polymerase (Bioline, London, UK), 0.5 μL of template DNA or 1st PCR product, and 2.9 μL of nuclease-free water, with a total reaction volume of 20 μL in a SimpliAmp Thermal Cycler (Thermo Fisher Scientific, Waltham, MA, USA). Thermal cycling consisted of an initial denaturation step at 94 °C for 10 min, followed by 35 cycles at 94 °C for 30 s, 55 °C for 60 s, and 72 °C for 1 min. The PCR products were visualised on 1.2% agarose gel stained with ethidium bromide. The expected products were 830 bp for the SSU rRNA and 870 bp for the *gp60* gene, respectively.

### DNA sequencing and species/subtypes identification

All PCR-positive samples were treated with ExoSAP-IT (Thermo Fisher Scientific) before sequencing to remove excess primers and dNTPs, according to the manufacturer’s instructions. PCR products were then analysed on a SeqStudio genetic analyzer using an ABI BigDye3.1 Terminator Cycle Sequencing Kit (Applied Biosystems, Carlsbad, CA, USA). The nucleotide sequences obtained for the SSU rRNA genes were then used to search the GenBank nucleotide database for any similarities using Basic Local Alignment Search Tool (BLASTn) software (National Center for Biotechnology Information, NCBI) for species identification. Top hit sequences with high similarity (>98%) were considered to be the assigned species. The *gp60* sequences were used for the assignment of the subtypes by counting the repeat number by ApE editor (by Davis M. Wayne; https://jorgensen.biology.utah.edu/wayned/ape/). The *gp60* sequences were aligned with the representative sequences of *Cryptosporidium* subtypes by using ClustalW, and a neighbour-joining tree with 1000 bootstrap replications was created using Molecular Evolutionary Genetics Analysis (MEGA) software version 7 [19]. The obtained sequences were deposited in GenBank (SSU rRNA of *C. parvum*; MH816914, MH816918; SSU rRNA of *C. hominis*; MH816925, MH816915; SSU rRNA of *C. felis*; MH816917; SSU rRNA of *C. meleagridis*; MT549157; *gp60* of *C. parvum*; MN904699–MN904725; *gp60* of *C. hominis*; MN904655–MN904698).

## Results

### Cryptosporidiosis prevalence among patients with diarrhoea at the UTH

Out of 19,033 faecal samples examined in the Parasitology Laboratory at UTH during the five-year period, 278 cases (1.46%) were microscopically positive for *Cryptosporidium* spp. Among the detected intestinal pathogenic parasites, *Cryptosporidium* spp. was the most prevalent parasite, followed by hookworm infection (159 cases: 0.84%) and *Giardia duodenalis* infection (149 cases: 0.78%) (Table S1). Of the 278 confirmed *Cryptosporidium* spp.-postive samples, 11 (3.9%) were mixed infections with other intestinal parasites: *Giardia duodenalis* (*n* = 3), *Strongyloides* sp. (*n* = 3), *Cystoisospora belli* (*n* = 2), hookworm (*n* = 1), *Blastocystis* sp. (*n* = 1) and *Endolimax nana* (*n* = 1).

In the analysed population, the highest cryptosporidiosis prevalence was in the 1–4 year age group (104/2762; 3.8%), followed by children less than 1 year (28/1182; 2.4%), and the 30–39 year age group (44/2922; 1.5%) (Table 1). Among the 1–4 year age group, infections were significantly more prevalent in males (69/104; 66.3%) than in females (35/104; 33.7%: *p* = 0.02). Conversely, a significant difference was observed in the 20–29 year age group, where the prevalence was higher among female patients (21/27; 77.8%) than in males (6/27; 22.2%) (*p* = 0.03).

**Table 1:** Age and sex distribution of cryptosporidiosis from 278 cases (*n* = 19,033) reported at the University Teaching Hospital, Lusaka, Zambia from January 2014 to May 2019.

Age group (years)	Positive samples *n* (% total number in the age group)	Female	Male	Total number of samples
<1	28 (2.4)	13	15	1182
1–4	104 (3.8)	35	69*	2762
5–9	5 (0.4)	4	1	1303
10–19	19 (1.0)	11	8	1941
20–29	27 (1.0)	21*	6	2675
30–39	44 (1.5)	26	18	2922
40–49	18** (0.9)	8	9	2070
50–59	7 (0.7)	4	3	1019
>60	5 (0.5)	1	4	1024
Unknown age	21** (1.0)	9	8	2135
Total	278	132	141	19,033

* Statistical significance between male and female prevalence was observed by Fisher’s exact test (*p* < 0.05).

** Sex information was missing for 4 patients and 1 patient in the “unknown age” and “40–49 year” categories, respectively.

**Figure 1:**
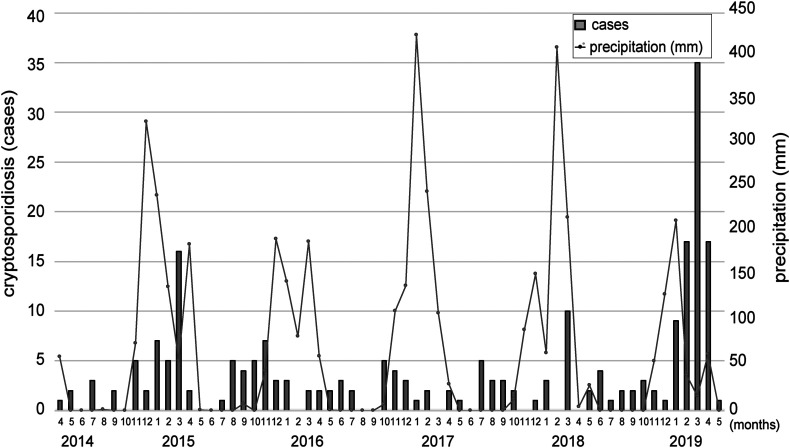
Monthly case number of cryptosporidiosis at the University Teaching Hospital (UTH, Lusaka, Zambia) over the data collection period (bar). Monthly average precipitations (mm) in Lusaka are also indicated on the right (line).

The confirmed cryptosporidiosis cases were also analysed by month in relation to the average monthly rainfall (mm) in Lusaka (Fig. 1). The number of cryptosporidiosis cases varied across time, with some months recording fewer than 10 cases. The highest prevalence was observed toward the end of the rainy seasons of 2015 and 2019. A notable increase in the number of patients with cryptosporidiosis was observed between January 2019 and April 2019. The number of patients with cryptosporidiosis was 5.6 times higher than what was recorded in the same period between 2015 and 2018 (78 cases vs. 14 average cases, respectively).

Information on age, HIV infection status, and the year of cryptosporidiosis detection were obtained, where available. Among the 51 cases with demographic data, the median age was 4 years (range: 4 months to 61 years). Thirty samples (58.8%) were from male patients and 15 samples (29.4%) were from HIV-positive patients. No significant associations between *Cryptosporidium* subtypes and patient age group, sex, year of cryptosporidiosis detection, or HIV infection status were observed.

### Sequencing results and phylogenetic tree analysis based on SSU rRNA and *gp60*

Seventy-one microscopically *Cryptosporidium*-positive samples stored from 2017 to 2019 were analysed for species and subtype identification using the SSU rRNA and *gp60* PCR, respectively. Out of 71 samples, 60 samples were successfully sequenced for SSU rRNA genes. Based on the SSU rRNA sequence analysis, one *C. felis* (GenBank: MH816917) and one *C. meleagridis* (MT549157) were obtained, and these two samples were not amplified by our *gp60* targeting primer. In all, 58 samples were amplified for both SSU rRNA and *gp60*, and species identified in SSU rRNA agreed with the *gp60* genotype families (genotype family I for *C. hominis* and genotype family II for *C. parvum*). Eleven samples were not amplified for the SSU rRNA gene, but the species could be identified based on the *gp60* sequence (Table 2).

**Table 2:** Species and subtype determination by sequence analysis of small subunit (SSU) rRNA and *gp60* loci of *Cryptosporidium* spp.

Species	*n* (%)	*gp60* subtype	*n* (%)
*C. hominis*	42 (59.2%)	IaA30R3	10 (14.1%)
		IaA27R3	2 (2.8%)
		IbA9G3	8 (11.3%)
		IdA21	2 (2.8%)
		IeA11G3T3	20 (28.2%)
*C. parvum*	27 (38.0%)	IIcA5G3a,b	12 (16.9%)
		IIeA12G1	11 (15.5%)
		IIeA11G1	3(4.2%)
		IIsA10G1	1(1.4%)
*C. felis*	1 (1.4%)	*gp60* not amplified	
*C. meleagridis*	1 (1.4%)	*gp60* not amplified	
Total samples	71		69

**Figure 2:**
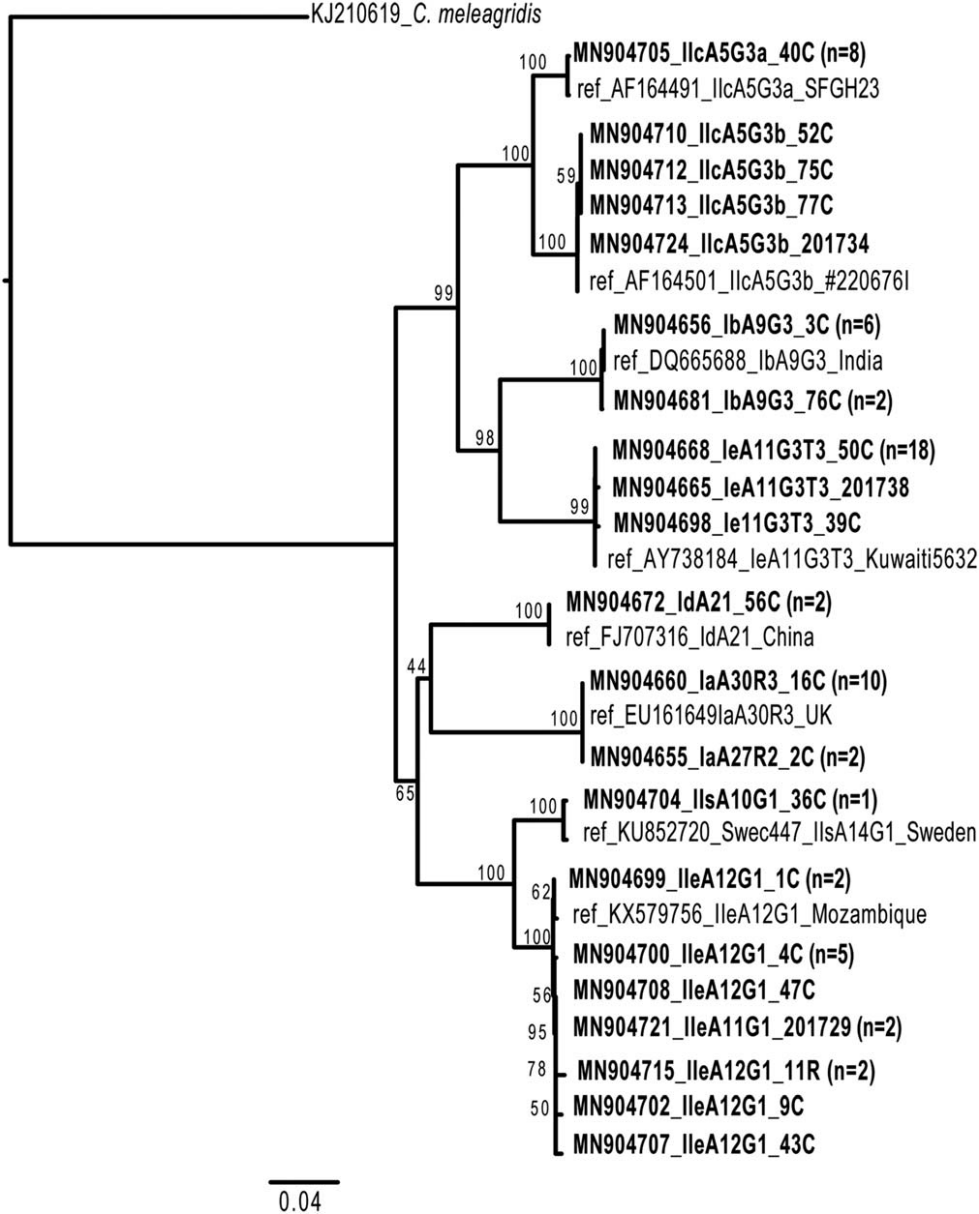
*gp60* phylogenetic tree. Phylogeny of *Cryptosporidium* spp. from human patients at the University Teaching Hospital (UTH) based on the partial *gp60* gene. The sequences determined in this study are indicated in bold font, and the GenBank number is shown. The representative sequence is shown if the same allele sequences were obtained, and the number of isolates is shown in parentheses. The neighbour-joining tree was constructed with 589 sites of the *gp60* gene using the Kimura-2 parameter. Bootstrap values with 1000 replicates are shown.

The identified *Cryptosporidium* species among the 71 isolates were: *C. hominis* (*n* = 42; 59.2%), *C. parvum* (*n* = 27; 38.0%), *C. felis* (*n* = 1; 1.4%), and *C. meleagridis* (*n* = 1; 1.4%). The most common *Cryptosporidium* subtype identified was IeA11G3T3 (*n* = 20; 28.2%) followed by IIcA5G3 (*n* = 12; 16.9%), IIeA12G1 (*n* = 11; 15.5%), and IaA30R3 (*n* = 10; 14.1%). In total, six genotype families, and nine subtypes; IaA30R3, IaA27R3, IbA9G3, IdA21, IeA11G3T3, IIcA5G3, IIeA12G1, IIeA11G1, and IIsA10G1, were identified in this study (Table 2). The obtained *gp60* sequences were compared with the representative sequence of each subtype family [9, 28] and/or the sequence obtained from the BLASTn top hit sequences. Although the species and subtypes of *Cryptosporidium* spp. observed in the studied population were diverse, the sequences within the same subtypes were relatively well-conserved as shown in the phylogenetic tree (Fig. 2). The sequences of the *C. hominis* IeA11G3T3, IaA27R3/IaA30R3, IdA21, and IbA9G3 subtypes showed homogeneous sequences, except for the TCA repeat number difference in the Ia subtype family (IaA27R3 and IaA30R3). Within the IIcA5G3 subtype, two subclades with diversity in the sequence of the 3′ region were observed. Eight and four isolates were clustered with the known subtypes IIcA5G3a and IIcA5G3b, respectively. All isolates from IIcA5G3a (*n* = 8) showed 100% identical sequences, while a few single nucleotide polymorphisms (1–2 SNPs) were observed within the IIcA5G3b subtype. The IIe subtype family also showed little diversity (1-3 SNPs), and subtypes with two TCA repeat numbers were detected (IIeA11G1, and IIeA12G1). The sequence from one isolate (36C; MN904704_IIsA10G1) was similar to the sequence deposited in GenBank from Sweden (KU852720) that was annotated as subtype IIsA14G1. Except for TCA repeat numbers, the sequences of isolates 36C and KU852720 were identical (Fig. 2).

## Discussion

*Cryptosporidium* was identified as the most common parasite among patients with diarrhoea diagnosed at the parasitology laboratory of the UTH, a tertiary-level hospital in Lusaka, Zambia during the studied period. The sequence analysis revealed that *C. hominis* and *C. parvum* were both common in the patients. Several studies have reported *Cryptosporidium* as a major cause of moderate-severe diarrhoea among young children in developing countries [18, 34]. In the current study, high *Cryptosporidium* prevalence was observed in the 1–4 year age group, emphasising the importance of *Cryptosporidium* as an aetiological agent of diarrhoea in this age group. When analysed by gender, the prevalence of cryptosporidiosis was significantly higher in males than in females in the 1–4 year age group. Similar findings were also documented in other countries including Nigeria, Guinea-Bissau, and Ghana [3, 7, 22], although the cause of this gender bias is unknown. In contrast, females in the 20–29 year age group were more likely to have cryptosporidiosis than males in our study. One possible explanation for the observed trend is the high risk of exposure to *Cryptosporidium* among women when caring for their infected children. According to Kimani et al., gender is one of the determinants of *Cryptosporidium* infection as women are more likely to become infected than men [16]. Although data on HIV positivity and immune status among our analysed population were not available, the higher prevalence of HIV in the adult female population in Zambia [5] may also explain the higher prevalence of *Cryptosporidium* in the female 20–29 year age group in the current study.

Analysis of *Cryptosporidium* cases by month revealed that the infection was more common during the rainy season. This observation corroborates the results of several studies in African countries that have shown similar seasonality [14, 33, 35]. Previous studies have reported an association between rainfall and diarrhoeal disease outbreaks [21, 31]. Similarly, we observed a higher number of cryptosporidiosis cases towards the end of the rainy season, which peaked in March for the years 2015, 2018, and 2019. This is possibly due to the contamination of water sources with human waste during flash flooding. However, despite having less precipitation in the year 2018/2019, more cases were recorded compared to those in the previous years. The high number of cases could have been attributed to water shortages causing people to consume contaminated water from unclean sources.

Although previous studies had reported high cryptosporidiosis prevalence in Zambia [15, 25], molecular studies on *Cryptosporidium* species or subtypes associated with the disease were not reported. Microscopic identification of *Cryptosporidium* at the species level is impossible as the species are morphologically similar, and hence can only be differentiated with molecular tools. The current study is the first to our knowledge to characterise *Cryptosporidium* species and subtypes among patients with diarrhoea in Zambia. We utilised SSU rRNA gene sequencing for species identification and *gp6*0 sequencing for subtyping of *C. parvum* and *C. hominis*. Although *C. parvum* and *C. hominis* are classified in distinct species for their biological and sequence differences [23], it is well known that some *gp60* alleles in these species are phylogenetically closely related [39]. Incongruence of the *gp60* loci topology was also observed in our analysed phylogenetic tree. A possible cause of the discordance may be recombination events between species and subtypes at the *gp60* locus [12, 20, 24]. The *gp60* subtypes are known to be associated with host tropism [24], virulence [4], and geographical distribution [11, 24], and thus provide useful information to understand the molecular epidemiology of cryptosporidiosis. In this study, of the four species detected, *C. hominis* was the most dominant species (59.2%). Subtype IeA11G3T3 was the most prevalent subtype (28.2%), with minimal sequence diversity. Three other subtypes of *C. hominis*, IaA30R3, IbA9G3, and IdA21, also showed low sequence variation within the analysed samples, suggesting that limited lineages of *C. hominis* were circulating in Lusaka. Subtype IIcA5G3 was the most prevalent (16.9%) in *C. parvum* and sequences were further classified into IIcA5G3a and IIcA5G3b. The IIcA5G3a subtype has been almost exclusively reported in humans [24]. A recent whole-genome analysis revealed that subtype IIc-a was clustered differently from zoonotic subtypes, and thus was referred to as *C. p. anthroponosum* compared to zoonotic subtypes *C. p. parvum*. It is speculated that *C. p. anthroponosum* resulted from recent genomic introgression events and is considered to be human-adapted *C. parvum* [24]. The documentation of the IIc-a subtype family in our study confirms earlier reports that it is mostly found in low-income countries and other African countries [10, 17, 35]. The IIc-b and IIe subtypes detected in the current study were also considered to be anthroponotic [36]. The major zoonotic subtype families IIa and IId have cosmopolitan distributions, with more cases being reported in industrialised countries [9, 11]. However, none of these zoonotic subtypes were detected in our study. In a neighbouring country, Malawi, four *C. hominis* subtypes (Ia, Ib, Id, and Ie), and two *C. parvum* subtypes (IIc and IIe) have been reported, and were all consistent with the subtype family identified in our study. Subtype IIs found in one sample in our study is a rare subtype that had been reported only from a human faecal sample in Sweden (GenBank KU852720). The host tropism, transmission cycle, and distribution of this subtype are largely unknown. Except for IIs, all subtypes of *C. parvum* and *C. hominis* detected in our study had also been reported in other African countries such as Ghana, Kenya, South Africa, or Nigeria [35]. Thus, like the cases in most of the other African countries, the human-to-human anthroponotic transmission route seems to be the major route of *Cryptosporidium* spp. infection in the urban setting of Zambia. We analysed samples from patients in urban areas with limited or no animal contact; hence, further studies in peri-urban and rural populations of Zambia where human-animal contact is high should be conducted to analyse the nation-wide epidemiological features of this disease. The detection of the potentially zoonotic species, *C. felis* and *C. meleagridis*, is a noteworthy finding that warrants further investigations, particularly as their transmission dynamics remain unknown [8, 9].

In our population, no associations between any particular subtype and the patient’s age, sex, year of cryptosporidiosis detection, or HIV status were observed. The high number of cryptosporidiosis cases observed in the 2018/2019 rainy season seems to be the result of infections originating from different sources. Since our sequence analysis targeted only patients with diarrhoea, further studies will be needed to assess the genetic heterogeneity of the *Cryptosporidium* parasite in non-diarrhoeal cases and animals in Zambia.

## Conclusions

Our study shows that *C. hominis* and *C. parvum* are the most common aetiological agents of cryptosporidiosis among diarrhoeal cases presenting at the UTH. The high prevalence in children (1–4 years) calls for more targeted interventions to control cryptosporidiosis in this age group. *Cryptosporidium hominis* subtypes Ia, Ib, Id, Ie, and *C. parvum* subtypes IIc, IIe and IIs were identified by sequencing for the first time in patients with diarrhoea in Zambia. Identification of the rare zoonotic species *C. felis* and *C. meleagridis* suggests that both anthroponotic and zoonotic transmission routes were responsible for cryptosporidiosis transmission in the studied population.

## Supplementary material

Supplementary material is available at https://www.parasite-journal.org/10.1051/parasite/2020050/olm Table S1 Intestinal parasite infections in patients attending the University Teaching Hospital (UTH, Lusaka, Zambia).

## Conflicts of interest

The authors declare that they have no conflict of interest.
